# Fertilizer source and mulch effects on nitrate‐N leaching and corn yield in irrigated sandy loam soil

**DOI:** 10.1002/jeq2.70226

**Published:** 2026-08-05

**Authors:** Swetabh Patel, Michael Kurtzhals, Arshdeep Singh, Amy M. Schmidt, Leslie Johnson, Javed Iqbal

**Affiliations:** ^1^ Department of Agronomy and Horticulture University of Nebraska‐Lincoln Lincoln Nebraska USA; ^2^ Haskell Agricultural Laboratory University of Nebraska‐Lincoln Concord Nebraska USA; ^3^ Department of Crop and Soil Sciences Oregon State University Corvallis Oregon USA; ^4^ Department of Biological System Engineering University of Nebraska‐Lincoln Lincoln Nebraska USA

## Abstract

Improving nitrogen (N) use efficiency while minimizing nitrate leaching remains a major challenge in irrigated sandy soils of the US Midwest. This 2‐year field study (2022–2023) in northeast Nebraska evaluated the effects of N source (inorganic fertilizer vs. beef manure) and surface mulch application on soil N dynamics, nitrate leaching, and corn (*Zea mays* L.) yield. Both growing seasons experienced below‐average precipitation, increasing reliance on irrigation and influencing N behavior in the soil profile. Early‐season pore water nitrate concentrations peaked in inorganic fertilizer treatments, especially without mulch, reflecting poor synchronization between N availability and crop uptake. In contrast, manure maintained lower and more stable nitrate concentrations, reducing leaching risk by up to 84%. Mulch further moderated nitrate levels, lowering concentrations by 14% in 2022 and 29% in 2023, although its effect was less pronounced when paired with manure. Seasonal nitrate leaching loads averaged 8.5 kg N ha^−^^1^ in inorganic treatments in 2022, compared to only 1.8 kg N ha^−^^1^ under manure. Agronomic responses indicated a tradeoff: inorganic fertilizers without mulch enhanced early growth, N uptake, and grain yield but at the cost of higher leaching potential. Conversely, manure and mulch improved soil N retention and reduced losses but slightly decreased yield and nitrogen use efficiency. Residual soil analyses confirmed that manure and mulch sustained more stable ammonium pools and minimized nitrate accumulation. These results highlight the importance of integrating manure and mulch into conventional inorganic nitrogen fertilizer management to balance agronomic performance and environmental protection in irrigated sandy soils vulnerable to nitrate leaching.

## Core Ideas

- Nitrogen source and mulch influenced soil nitrate dynamics in irrigated sandy soils.
- Manure reduced nitrate leaching by 80% compared to inorganic fertilizer across two growing seasons.
- Inorganic fertilizer increased yield but also raised nitrate leaching risk under irrigation.
- Mulch application moderated nitrate peaks and improved nitrogen retention in the soil.
- Integrated nitrogen fertilizer and mulch management can enhance nitrogen use efficiency and reduce losses.

## Plain Language Summary

Managing nitrogen (N) in irrigated sandy soils is challenging because water can quickly carry nitrate below the root zone, increasing the risk of groundwater contamination. This study evaluated how different N sources, manure versus inorganic fertilizer, and the use of surface mulch affect N movement, crop uptake, and yield in irrigated corn. Over 2 years, manure reduced nitrate leaching up to 84% compared to inorganic fertilizer, especially when combined with mulch. While inorganic fertilizer improved corn yield, it also increased the risk of N loss. Mulch helped slow N movement in the soil but had a limited impact when used with inorganic fertilizer alone. These findings suggest that using manure along with mulch may offer a practical way to balance crop production with protecting water quality in sandy, irrigated cropping systems.

**Abbreviations**
BGMA: Bazile Groundwater Management Area
BMPs: best management practices
ET: evapotranspiration
HI: harvest index
NDRE: normalized difference red edge index
NUE: nitrogen use efficiency
PFP: partial factor productivity
SDI: subsurface drip irrigation
SPAD: soil plant analysis development

## 1 INTRODUCTION

Nitrogen (N) management in irrigated cropping systems is a critical agronomic and environmental challenge in the US Midwest. Excessive N application, particularly in coarse‐textured, irrigated soils, has contributed to widespread nitrate contamination of groundwater, a major drinking water source in rural regions. Nitrate is highly mobile in soil and readily transported with water, especially under irrigation where excess water accelerates downward movement through sandy profiles (Power & Schepers, 1989; Rabalais et al., 2002). This issue has serious implications for public health, agricultural sustainability, and water resource management. Nebraska is among the most intensively farmed and irrigated states in the United States., with approximately 17.8 million ha (44 million acres) of farmland, nearly 3.2 million ha (7.96 million acres) under irrigation, and >4.3 million ha (10.75 million acres) planted to corn in 2025 (USDA‐NASS, 2024). Groundwater supplies drinking water for approximately 85%–90% of the state's population, making it highly vulnerable to nitrate contamination (Exner et al., 2014; NDEQ, 2018; Puckett et al., 2011).

As the dominant crop in the state, corn (*Zea mays* L.) has a high N demand, often exceeding 150–200 kg N ha^−^^1^ depending on yield goals and environmental conditions (Ferguson, 2015; Randall & Mulla, 2001). The combination of high N inputs, irrigated production systems, and coarse‐textured soils increases the potential for nitrate leaching below the root zone (Singh et al., 2023). Numerous studies have linked elevated surface and groundwater nitrate levels to the long‐term use of synthetic N fertilizers in agricultural production systems (Exner et al., 2014; Keeney & DeLuca, 1993; Singh et al., 2025b; Singh et al., 2025a; Spalding & Exner, 1993). In regions such as the Bazile Groundwater Management Area in northeast Nebraska, nitrate concentrations have increased substantially due to increased use of inorganic fertilizers, prompting regulatory interventions and costly mitigation efforts (Galloway et al., 2008; Kross et al., 1993; NDEQ, 2018). Similar patterns have been observed across the Midwest, particularly in regions characterized by high nitrogen fertilizer inputs, continuous corn or corn–soybean rotations, and intensive production systems on vulnerable soils (David et al., 2010; Galloway et al., 2008; Jaynes et al., 2001; Randall & Mulla, 2001; Robertson & Vitousek, 2009). Nitrate is highly mobile in soil and readily transported with water, especially under irrigation where excess water accelerates downward movement through sandy profiles (Power & Schepers, 1989; Rabalais et al., 2002).

Improving nitrogen management in these systems is central to the 4R nutrient stewardship framework, which emphasizes the application of the right source, rate, time, and placement of nutrients to maximize crop productivity while minimizing environmental losses (Fixen, 2007; IPNI, 2012; Johnston & Bruulsema, 2014). This framework promotes synchronization of nitrogen supply with crop demand and site‐specific conditions, which is particularly critical in irrigated sandy soils where low water‐holding capacity and high permeability increase nitrate mobility and leaching risk (Di & Cameron, 2002; Randall & Mulla, 2001). Under such conditions, management decisions related to fertilizer source and residue management play a key role in controlling nitrogen transformations, availability, and transport within the soil–plant system.

Irrigation method further influences nitrogen dynamics by altering water movement and nitrate transport pathways. While center pivot irrigation is the dominant system in Nebraska, subsurface drip irrigation (SDI) is a less common but increasingly adopted technology in high‐value cropping and research systems (Colaizzi et al., 2019; Lamm et al., 2012). The SDI delivers water directly to the root zone through buried drip lines, resulting in more localized wetting patterns, reduced surface evaporation, and minimal runoff (Ayars et al., 1999; Camp, 1998). However, this concentrated wetting front can alter nitrate distribution and transport compared to sprinkler systems, potentially affecting leaching dynamics and nitrogen availability (Ajdary et al., 2007; Hanson et al., 2009; Phene et al., 1991). Therefore, understanding nitrogen behavior under SDI is important for interpreting results and translating findings to broader irrigated systems.

To mitigate nitrate losses, research has focused on best management practices (BMPs) that improve nitrogen use efficiency (NUE) and better synchronize N supply with crop demand. These practices include the strategic use of organic amendments such as manure and the carbon‐based materials like mulch (Olivo et al., 2024; Van Es & Karlen, 2019). Manure is typically applied preplant or early in the growing season to provide a gradually mineralizing nitrogen source that aligns more closely with crop uptake. Mulch is applied to the soil surface to moderate soil temperature, conserve moisture, and reduce evaporation, thereby influencing nitrogen transformation and movement. Manure, in particular, is an attractive option as it provides both an immediate and graduate release of N, offering improved synchronization with crop uptake and reducing leaching risk (Magdoff & van Es, 2000; Sarrantonio & Scott, 1988). Despite Nebraska's large livestock industry, manure remains underutilized in many cropping systems (Risse et al., 2006). Mulch can further enhance soil water conservation and moderate soil conditions, thereby influencing N dynamics (Cambardella et al., 2015).

Although individual BMPs have been widely studied (Dinnes et al., 2002; Gentry et al., 2009; Randall et al., 2008), limited information exists on their combined effects under irrigated sandy soil conditions. In particular, interactions among manure and mulch may influence soil nitrogen dynamics, nitrate leaching, and crop productivity in ways that are not captured when these practices are evaluated independently (Dinnes et al., 2002; Gentry et al., 2009; Randall et al., 2008). This interferes with our ability to develop integrated nitrogen management strategies that balance productivity with environmental protection.

Given the increasing regulatory and economic pressures associated with nitrate contamination in Nebraska, there is a need to identify management systems that optimize nitrogen use while minimizing environmental losses. This is especially important in regions where coarse‐textured soils and irrigation amplify leaching potential.

The objective of this study was to evaluate the effects of manure and inorganic fertilizer, both applied with and without woodchip mulch, on soil N dynamics, nitrate leaching, and corn productivity in irrigated sandy soils of northeast Nebraska. By assessing these practices individually and in combination under SDI, this research aims to inform integrated N management strategies that improve NUE while protecting groundwater quality in Nebraska and similar agroecosystems.

**Core Ideas**

- Nitrogen source and mulch influenced soil nitrate dynamics in irrigated sandy soils.
- Manure reduced nitrate leaching by 80% compared to inorganic fertilizer across two growing seasons.
- Inorganic fertilizer increased yield but also raised nitrate leaching risk under irrigation.
- Mulch application moderated nitrate peaks and improved nitrogen retention in the soil.
- Integrated nitrogen fertilizer and mulch management can enhance nitrogen use efficiency and reduce losses.

## 2 MATERIALS AND METHODS

### 2.1 Site description

This study was conducted in 2022 and 2023 at the Haskell Agricultural Laboratory of the University of Nebraska–Lincoln near Concord, NE (42.394° N, 96.950° W; elevation 438 m). Both years used the same research field, classified as Blendon fine sandy loam (0%–2% slopes, well‐drained) (Soil Survey Staff, 2023). The site has a sub‐humid climate and was managed under no‐till with SDI. The SDI was used to provide precise water delivery directly to the root zone and to better evaluate nitrogen dynamics under controlled irrigation conditions. Compared to sprinkler systems, SDI creates localized wetting patterns with predominantly vertical water movement, which can influence nitrate transport and leaching behavior in sandy soils (Ayars et al., 1999). Prior to study initiation, baseline soil properties were determined by collecting six soil cores (0‐ to 20‐cm depth) from each experimental block. Cores were composited within each block and submitted to Ward Laboratories for analysis (see Table S1). Weather data during the study period (2022–2023), as well as long‐term (20‐year) climatic data (2012–2021), were obtained from the nearest weather station located in Concord, NE (Singh et al., 2021).

### 2.2 Experimental design and treatments

The study included 24 plots (6.10 × 30.48 m), planted to corn in both years (Table S2). Treatments followed a factorial design of two N sources (beef manure and inorganic fertilizer) and two mulch levels (woodchip mulch and no mulch). This arrangement produced four combinations: (1) MM: manure with mulch, (2) MNM: manure without mulch, (3) IFM: inorganic fertilizer with mulch, and (4) IFNM: inorganic fertilizer without mulch.

All the treament plots within each year received the same total N rate (Table S2), calculated using the University of Nebraska–Lincoln's N recommendation tool (Iqbal et al., 2023), which considered irrigation‐derived N and credits from prior manure. At planting, 30% of the N was applied uniformly as urea (46‐0‐0) using a ground‐driven mobility dry fertilizer spreader with a 50‐ft swath. For manure treatments, the remaining 70% of N was applied at planting as solid beef manure using a Gehl 250 manure spreader (Gehl Company) pulled by a John Deere 5425 tractor (John Deere). Manure samples were analyzed prior to application, and rates were set using UNL guidelines (Shapiro et al., 2021). For inorganic fertilizer treatments, the remaining 70% of N was applied at the V6 corn stage (Abendroth et al., 2011) as urea–ammonium nitrate (UAN; 28‐0‐0). The use of different fertilizer sources between planting and sidedress reflects standard agronomic practices aimed at improving NUE by synchronizing nitrogen availability with crop demand. In addition, inorganic plots received supplemental phosphorus (P), sulfur (S), and zinc (Zn) at planting to align with nutrient inputs from manure. Woodchip mulch (mixed hardwood species) was randomly applied to half of the manure and inorganic plots at planting using a manure spreader.

In 2022, a 111‐day relative maturity corn hybrid P1108Q, was planted, while in 2023 a 103‐day hybrid P0339AM, was used after a planting error on the initial seeding date required corn to be re‐seeded (Table S2). Hybrids were no‐till seeded at 81,000 seeds ha^−^^1^. Field operations varied slightly: in 2022 corn was planted first, with manure and mulch applied afterward; in 2023 manure and mulch were applied first, followed by planting with a row‐cleaner‐equipped planter. SDI was managed based on soil moisture, with irrigation triggered when soil water content approached 50%–60% of field capacity to meet crop water demand. A detailed schedule of field operations for both years is provided in Table S2.

### 2.3 Soil sampling and analysis

Deep soil cores (0–120 cm) were collected before planting in spring and after harvest in fall of each year. Three random cores per plot were extracted with a truck‐mounted Giddings probe (Giddings Machine Company) with 4.1 cm internal diameter and segmented into four depth increments (0–30, 30–60, 60–90, and 90–120 cm). Samples from the same depth within each plot were composited, cooled, and transported for laboratory analysis.

To monitor in‐season N dynamics, additional 0–30 cm samples were collected monthly in June, July, and August 2023. Six random cores per plot were taken using a hand‐push Dakota probe (Dakota Peat & Equipment) and composited. All samples were extracted with 2 M KCl and analyzed colorimetrically for nitrate‐N (NO_3_^−^‐N) and ammonium‐N (NH_4_^+^‐N) (Hood‐Nowotny et al., 2010; Kandeler & Gerber, 1988). Nitrate and ammonium concentrations were converted to a mass basis using measured, depth‐specific soil bulk densities (Singh & kukal, 2024).

### 2.4 Plant growth parameters

Plant height was measured at V10 (± 1) from the base to the extended tip of the tallest leaf. For each plot, mean plant height was calculated from 10 randomly selected plants from the center two rows to avoid the border effects. Leaf chlorophyll was measured using a SPAD (soil plant analysis development) meter (Holland Scientific) at V10 and R1, with readings taken from the topmost leaf with open collar at V10, and the leaf below the primary ear at R1. Twenty plants per plot were sampled and averaged. Canopy development was evaluated at V10 and R1 using the normalized difference red edge index (NDRE), measured with a RapidSCAN CS‐45 canopy sensor (Holland Scientific) held 0.5 m above the canopy (Patel et al., 2019).

### 2.5 Crop yield and NUE

Corn grain yield was determined from the center four rows of each plot using a John Deere 9560 small plot combine. Yields were adjusted to 15.5% moisture and reported as Mg ha^−1^. To complement mechanical harvest, six consecutive plants per plot were hand‐harvested for biomass. Ears and stalks were separated and weighed fresh. Stalks were shredded using a Vermeer 303 woodchipper (Vermeer 303, Vermeer Corporation) on a John Deere 5425 tractor, and subsamples of both stover and ears were oven‐dried at 71°C to determine dry matter. After drying, ears were shelled, and grain weight was recorded.

Harvest index (HI) was calculated as the ratio of grain yield to total aboveground biomass. Subsamples of dried grain and stover were analyzed at Ward Laboratories (Kearney, NE) for total N content. These data were used to estimate total plant N uptake and calculate NUE indicators, including the nitrogen harvest index (NHI) and partial factor productivity (PFP):

(1) $$\mathrm{NHI}\;\;\left(\%\right)=\frac{\mathrm{Grain}\;N}{\mathrm{Total}\;\mathrm{plant}\;N\;\mathrm{uptake}}\;\times \;100$$

(2) $$\mathrm{PFP}\;\left(\mathrm{kg}\;\mathrm{grain}\;\mathrm{kg}\;N^{-1}\right)=\frac{\mathrm{Grain}\;\mathrm{yield}}{N\;\mathrm{applied}}\;$$

The NHI indicates the proportion of total plant nitrogen allocated to grain, providing insight into nitrogen partitioning and utilization efficiency within the crop. While the PFP represents the amount of grain produced per unit of applied nitrogen and serves as an integrative measure of NUE, reflecting both fertilizer effectiveness and overall system performance.

### 2.6 Lysimeter installation, water sampling, and nitrate leaching

Soil pore water NO_3_^−^‐N and NH_4_^+^‐N concentrations were monitored using suction cup lysimeters. Two ceramic cups per plot were installed at 1.2‐m depth, spaced 6 m apart between the center rows (Singh et al., 2025a; Venterea et al., 2011). A vacuum of 70–80 kPa was applied with a manual pump, and water was collected the following day. Samples were collected 1–2 times weekly, particularly after rainfall or irrigation. In 2022, sampling ran from April 11 to October 6 (22 events); in 2023, from May 18 to August 30 (21 events). Collected samples (10–15 mL) were acidified with 100 µL of 0.1 N HCl, stored in coolers, and transported to the laboratory.

Nitrate leaching load (kg N ha^−^^1^) was calculated using a water balance approach coupled with lysimeter data (Maharjan et al., 2014). Daily drainage (D) was estimated using the following equation (Djaman & Irmak, 2013):

(3) D=P+I−R−ET−ΔS.

where *P* is precipitation, *I* irrigation, *R* runoff, ET evapotranspiration, and *ΔS* the change in soil water storage within 0–120 cm. Evapotranspiration was estimated as alfalfa crop coefficient (Kc) × reference ET using the Penman–Monteith equation (Allen et al., 1998; 2000), with adjustments for corn growth stage (Lo et al., 2019). Soil moisture confirmed that drainage occurred only when storage exceeded field capacity. Daily nitrate leaching was calculated by multiplying drainage by interpolated NO_3_^−^‐N concentrations. A conversion factor of 0.01 converted ppm to kg ha^−^^1^ (Pawlick et al., 2019). Seasonal cumulative leaching load was the sum of daily leaching values.

### 2.7 Statistical analysis

All statistical analyses were performed using the generalized linear mixed model procedure (PROC GLIMMIX) in SAS 9.4 (SAS Institute Inc.). Because significant year × treatment interactions were observed for several response variables, data were analyzed separately by year.

Temporal changes in soil pore water NO_3_^−^‐N and residual soil NO_3_^−^‐N and ammonium‐N (NH_4_^+^‐N) concentrations (spring and fall) were evaluated using a two‐way repeated measures analysis of variance (ANOVA) with an autoregressive error structure. In these models, treatment was specified as a fixed effect, sampling time or stage as the repeated factor, and replication and its interactions as random effects. Other response variables, including grain yield, PFP, HI, plant and grain N uptake, grain N concentration, NHI, plant height, NDRE, SPAD readings, and nitrate leaching load, were analyzed using one‐way ANOVA, with treatment as a fixed effect and replication as a random effect.

Pairwise contrasts were used to compare (i) manure versus inorganic fertilizer and (ii) mulch versus no mulch. Means were separated using least square means, and differences were considered significant at *p* < 0.05.

## 3 RESULTS AND DISCUSSION

### 3.1 Weather conditions

**FIGURE 1:**
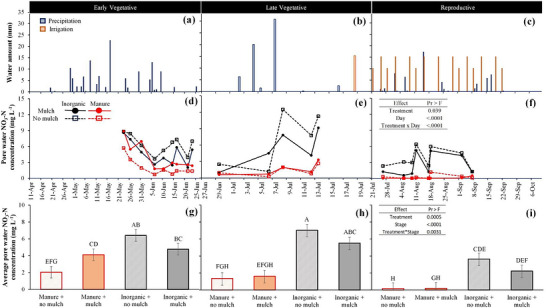
Effect of treatments on daily (d–f) and sub‐seasonal average (g–i) pore water nitrate concentration for 2022, Concord, NE. Water amount (a–c) for the early vegetative, late vegetative, and reproductive phases is shown. Precipitation and irrigation are shown in blue and orange colors, respectively. The *p*‐value tables for two‐way repeated measures analysis of variance (ANOVA) results are shown for daily (f) and sub‐seasonal averages (i). The different uppercase letters above bars represent significant differences among treatments within each panel (g–i).

**FIGURE 2:**
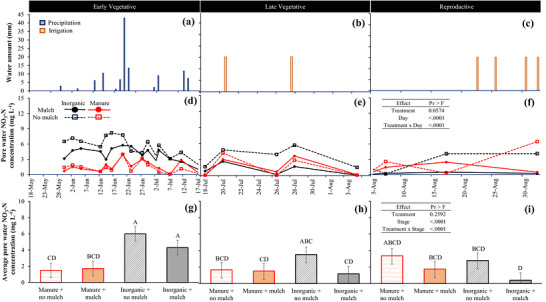
Effect of treatments on daily (d–f) and sub‐seasonal average (g–i) pore water nitrate concentration for 2023, Concord, NE. Water amount (a–c) for the early vegetative, late vegetative, and reproductive phases is shown. Precipitation and irrigation are shown in blue and orange colors, respectively. The *p*‐value tables for two‐way repeated measures analysis of variance (ANOVA) results are shown for daily (f) and sub‐seasonal averages (i). The different uppercase letters above bars represent significant differences among treatments within each panel (g−i).

Weather conditions in 2022 and 2023 were considerably drier than the long‐term (2012–2021) average (Figure S1). From April to October 2022, cumulative precipitation totaled 265 mm, less than half the historical average of 581 mm. In 2023, precipitation from May to October was 251 mm, also well below the corresponding average of 515 mm. Rainfall in 2022 was relatively well distributed, though still below normal in most months. In contrast, 2023 precipitation was concentrated in May and June (210 mm combined), with no rainfall recorded from August through October. This late‐season dryness increased reliance on irrigation during reproductive and grain‐filling stages. Total seasonal water input, combining rainfall and irrigation, was similar between years, 478 mm in 2022 and 464 mm in 2023 (Figures 1 and 2). These below‐normal precipitation levels, particularly the dry late season in 2023, likely influenced N dynamics and nitrate movement, as irrigation became the dominant driver of water and solute fluxes.

### 3.2 Pore‐water nitrate concentration and leaching load

#### 3.2.1 Pore‐water NO_3_^−^‐N concentration

Because this study was conducted under SDI, nitrate movement was primarily governed by localized soil moisture dynamics within the root zone rather than surface‐driven processes typical of sprinkler or rainfed systems. This distinction is important when interpreting treatment effects and comparing results with previous studies.

In 2022, early‐season nitrate spikes (up to 12.3 mg L^−1^ NO_3_^−^‐N) were observed across all treatments, likely due to rapid N mineralization combined with rainfall and irrigation under low N uptake conditions, consistent with Ma et al. (2021) and Singh et al. (2024). Similar early‐season nitrate accumulation has been widely reported in rainfed and tile‐drained systems in the Midwest, where fertilizer‐derived nitrate exceeds crop demand during early growth (Jaynes et al., 2001; Randall & Mulla, 2001). Under SDI, however, these effects may be moderated due to localized water application and reduced surface wetting, which can limit widespread nitrate movement compared to sprinkler or tile‐drained systems. Nevertheless, in coarse‐textured soils, vertical water flux within the wetted zone can still facilitate downward nitrate transport when nitrogen supply exceeds crop demand. Nitrate concentrations declined later in the season (0.1–6.2 mg L^−^^1^ NO_3_^−^‐N), reflecting crop uptake and movement below the root zone (Figure 1d–f). Inorganic fertilizer plots, especially without mulch (IFNM), maintained significantly higher NO_3_^−^‐N concentrations through the season, particularly following the V6 sidedress N application. This pattern indicates poor synchronization between fertilizer N release and crop demand, consistent with observations under rainfed and tile‐drained systems in the Midwest (Randall & Mulla, 2001). Sub‐seasonal averages confirmed higher pore‐water NO_3_^−^‐N in inorganic fertilizer treatments compared with manure treatments (Figure 1g–i).

Similar trends were observed in 2023, although magnitude varied with precipitation distribution. Inorganic fertilizer plots again showed early‐season nitrate peaks, reaching up to 8 mg L^−^^1^, while manure plots maintained lower and more stable concentrations likely due to gradual nitrogen release better synchronized with crop demand (Figure 2d–f). Elevated NO_3_^−^‐N concentrations in IFNM persisted into the late vegetative stage, consistent with findings from sprinkler‐irrigated systems wherein in‐season fertilizer applications increase nitrate mobility under moist conditions (Randall & Vetsch, 2005). Mulch reduced nitrate concentrations relative to non‐mulched treatments, particularly in 2023, likely due to moderation of soil temperature and moisture and delayed N mineralization (Gentry et al., 2009).

**FIGURE 3:**
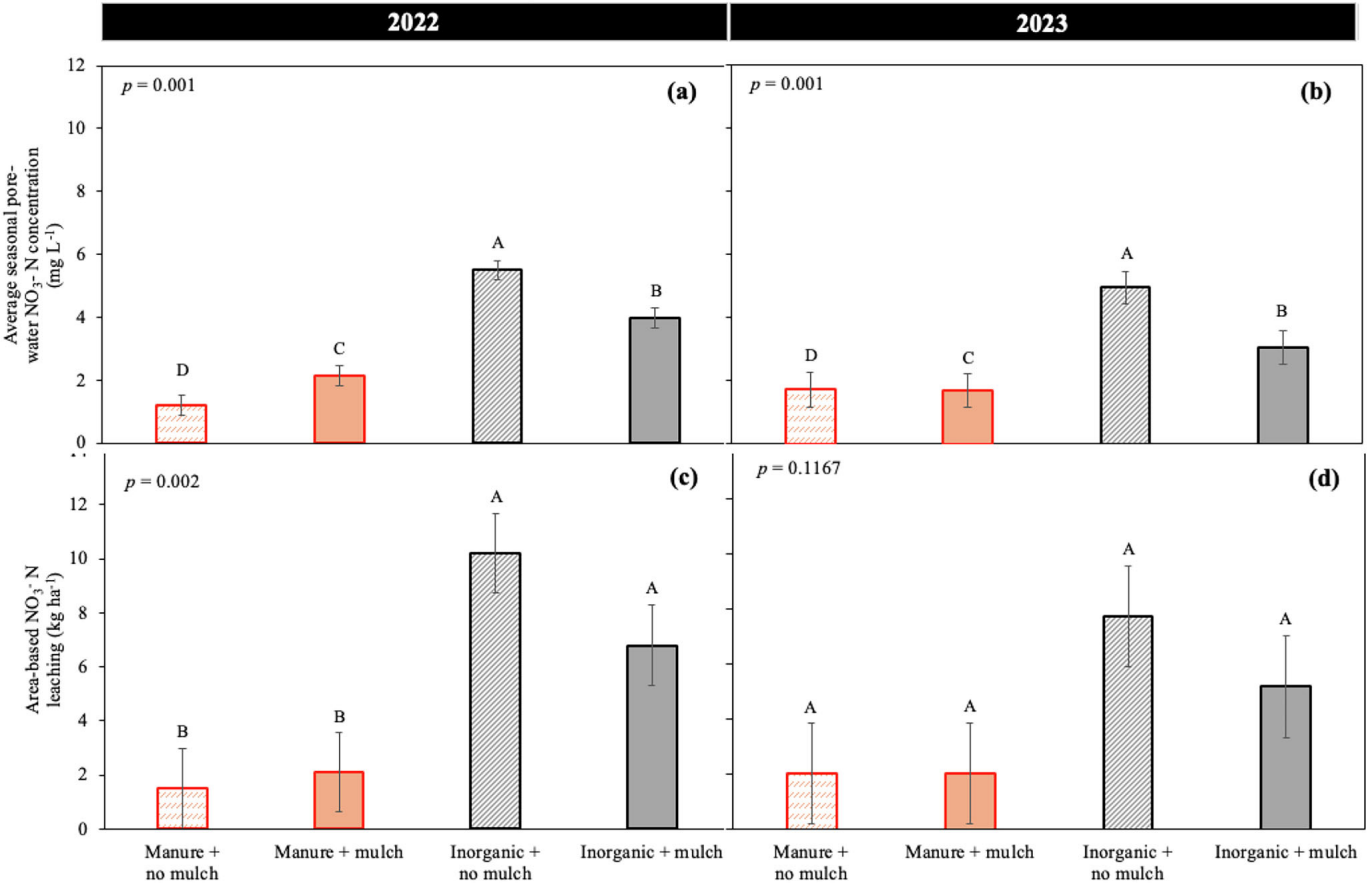
Effect of treatments on average seasonal porewater NO_3_‐N concentrations (a and b) and nitrate leaching load (c and d) in 2022 and 2023, Concord, NE. The different uppercase letters above bars represent significant differences among treatments within each panel.

Across both years, inorganic fertilizer treatments resulted in significantly higher seasonal NO_3_^−^‐N concentrations, approximately three times greater than manure in 2022 and twice as high in 2023 (Figure 3a,b). Mulch reduced nitrate concentrations by 14% in 2022 and 29% in 2023, with the greatest reduction observed in MM. The IFNM treatment consistently exhibited the highest NO_3_^−^‐N concentrations (6.0–6.5 mg L^−^^1^), whereas manure treatments maintained the lowest levels (1.5–2.0 mg L^−^^1^). These results are consistent with previous studies demonstrating that inorganic fertilizers increase nitrate availability and leaching risk, while organic amendments reduce nitrate losses through gradual mineralization and improved soil water retention (Cambardella et al., 2015; Gentry et al., 2009). However, the magnitude of differences observed in this study is lower than many rainfed or sprinkler‐based systems, likely due to the interaction of SDI with sandy soils, where localized water application and reduced surface wetting can limit widespread nitrate movement compared to sprinkler or tile‐drained systems. Overall, inorganic fertilizers, especially without mulch, promote nitrate accumulation and leaching potential, whereas manure improves nitrogen synchronization and reduces environmental risk.

#### 3.2.2 Nitrate leaching load

Seasonal nitrate leaching loads followed patterns observed in pore‐water NO_3_^−^‐N concentrations. In 2022, inorganic fertilizer treatments resulted in significantly greater leaching losses (8.5 kg N ha^−^^1^) compared with manure treatments (1.8 kg N ha^−^^1^) (Figure 3c). In 2023, treatment effects were not statistically significant; however, manure treatments consistently exhibited numerically lower nitrate leaching losses than inorganic fertilizer treatments (Figure 3d). Similar reductions in nitrate leaching under manure‐based systems have been reported in both rainfed and irrigated environments (Jaynes et al., 2001; Randall & Mulla, 2001). Mulch provided modest reductions in nitrate leaching, but its effect was smaller than that of nitrogen source. Overall, these results confirm that nitrogen source was the primary driver of nitrate leaching under SDI conditions, with inorganic fertilizer treatments, particularly without mulch, resulting in greater losses than manure‐based systems. These findings highlight the importance of nitrogen source selection for minimizing leaching losses in SDI‐based production systems.

### 3.3 In‐season soil nitrogen dynamics

**FIGURE 4:**
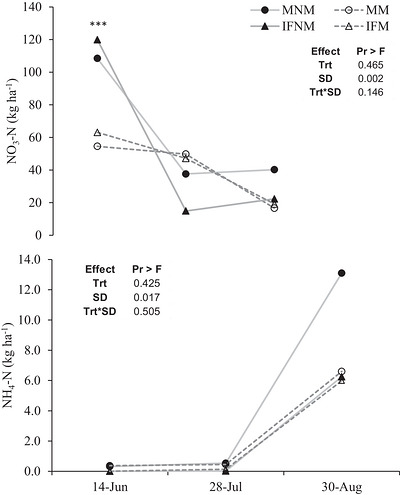
In‐season soil NO_3_^−^‐N (upper panels) and NH_4_^+^‐N (lower panels) availability in soil sampled at 0‐ to 30‐cm depth for 3 months (June, July, and August) of 2023. Asterisks indicate significant mean comparison between manure and inorganic fertilizer with mulch (MM + IFM) and manure and inorganic fertilizer without mulch (MNM + IFNM) at each sampling date as determined by least‐square means (*p ≤ 0.05, **p ≤ 0.01, and ***p ≤ 0.0001). Significant *p* values using repeated measure generalized linear mixed model procedure (SAS) (PROC GLIMMIX) for the differences of fertilizer treatment (Trt) and sampling date (SD) are shown.

In 2023, soil NO_3_^−^‐N concentrations (0–30 cm) showed pronounced temporal variability, with notable treatment differences early in the season (Figure 4). At the early vegetative stage (June 14), NO_3_^−^‐N levels were nearly double in MNM and IFNM compared with MM and IFM, indicating lower nitrate availability in mulched treatments. This pattern suggests that mulch likely reduced early nitrate availability through temporary N immobilization associated with the high C:N ratio of woodchips, a mechanism consistent with previous studies in similar systems (Randall & Mulla, 2001). As the season progressed, soil NO_3_^−^‐N concentrations declined across all treatments, reflecting increased crop N uptake and redistribution of nitrate within the soil profile. This decline aligns with the reduced pore‐water nitrate concentrations and leaching trends described earlier, indicating that early‐season nitrate availability strongly influenced subsequent nitrogen movement and losses.

In contrast to nitrate, soil NH_4_^+^‐N concentrations remained low early in the season across all treatments (Figure 4), indicating rapid nitrification under favorable conditions. However, by the late vegetative stage (July 28), NH_4_^+^‐N concentrations increased markedly in the IFM treatment, suggesting delayed nitrification of readily available inorganic N under mulched conditions. In contrast, this pattern was not observed in MM, likely due to the gradual mineralization of organic N, which distributes ammonium release over time and prevents short‐term accumulation. Reduced soil temperature and microbial activity under mulch may further contribute to slower ammonium conversion to nitrate (Greenly & Rakow, 1995). By the reproductive stage (August 30), NH_4_^+^‐N concentrations stabilized across treatments, indicating that most applied N had either been taken up by the crop or transformed into nitrate.

Together, these dynamics reinforce earlier observations that nitrogen availability and movement are closely linked to nitrogen source and mulch effects under SDI.

Overall, the combined evidence from soil nitrogen dynamics, pore‐water nitrate concentrations, and leaching losses demonstrates that early‐season nitrogen availability plays a critical role in determining both agronomic efficiency and environmental outcomes. Treatments that moderated early nitrate availability, particularly manure and mulch, improved synchronization between nitrogen supply and crop demand, thereby reducing the risk of nitrate loss (Gentry et al., 2009).

### 3.4 Plant growth parameters

Consistent with the soil nitrogen dynamics and pore‐water nitrate patterns, plant growth responses were strongly influenced by nitrogen source and mulch application (Table 1). Early‐season differences in nitrogen availability and soil microclimate translated directly into variations in plant height, chlorophyll content (SPAD), and canopy development (NDRE).

**TABLE 1:** Treatment means and significance for plant height, soil plant analysis development (SPAD) (chlorophyll content), and NDRE (normalized difference red edge index) readings as affected by treatments at V10 and R1 corn growth stage in 2022 and 2023, Concord, NE.

	2022					2023				
Source of effects	V10			R1		V10			R1	
	Height (cm)	SPAD	NDRE	SPAD	NDRE	Height (cm)	SPAD	NDRE	SPAD	NDRE
MNM	54a	44a	0.381a	55	0.412b	144a	54a	0.354a	57a	0.349
MM	45b	38b	0.314c	54	0.412b	121b	50b	0.324b	52b	0.341
IFNM	51ab	41ab	0.393a	56	0.417ab	122b	55a	0.348a	57a	0.336
IFM	45b	33c	0.332b	56	0.427ab	124b	49b	0.294c	55a	0.34
**Contrasts**	*P* > *F*									
Manure vs inorganic	NS	*	*	NS	*	NS	NS	***	NS	NS
Mulch vs. No mulch	**	***	***	NS	NS	NS	***	***	***	NS

*Note*: MNM and MM indicate manure treatments without and with mulch, respectively; IFNM and IFM indicate inorganic fertilizer treatments without and with mulch, respectively. Treatment means followed by the same letter within a column are not significantly different. Columns that do not contain letters indicate no significant difference between treatments.

Abbreviation: NS, not significant.

*, **, and ***: Significant at *p* < 0.05, 0.01, and 0.001, respectively.

At the V10 stage in 2022, corn plants in mulched plots were 14%–17% shorter than those in non‐mulched plots. Plant height in the MNM (54 cm) was 17% greater than in the MM (45 cm) treatment, while plants in the IFNM treatment (51 cm) were 13% taller than those in the IFM (45 cm) treatment. These reductions align with the lower early‐season nitrate availability observed in mulched treatments, as well as field conditions where manure and mulch applied after planting created a surface layer that impeded emergence and reduced early growth. Mulch also likely lowered soil temperature, slowing germination (Blanco‐Canqui & Lal, 2009; Teasdale & Mohler, 2000). By the R1 stage, height differences disappeared, likely due to compensatory growth, where improved soil temperature and increased nitrogen availability later in the season enhanced growth rates, allowing plants to recover from early delays (Andrade et al., 1999; Tollenaar & Lee, 2002).

In 2023, overall plant height was greater than in the 2022, although mulch again reduced early‐season growth. At the V10 stage, plants in the MNM treatment (144 cm) were 19% taller than in the MM (121 cm) treatment, whereas no significant differences were observed between the IFM and IFNM treatments. The greater mulch effect under manure is consistent with delayed nitrogen availability observed earlier, where mulch slowed mineralization of organic N. Even though manure and mulch were applied before planting with row cleaners, residual woodchips from 2022 combined with new mulch may have created excessive residue, further suppressing early development. As in 2022, these differences were not significant at R1, indicating recovery as nitrogen availability improved over time.

SPAD readings at V10 mirrored plant height responses and reflected early‐season nitrogen status consistent with pore‐water and soil nitrate trends. In both years, MNM and IFNM had 10%–20% higher values than mulched treatments, reflecting greater early N availability in no‐mulch systems. By R1, SPAD values converged across treatments, except that MM continued to show lower readings in 2023, consistent with delayed nitrogen release observed in soil N dynamics. This delay likely resulted from mulch‐induced microbial immobilization and reduced soil temperature, both of which can slow mineralization of organic N from manure (Blanco‐Canqui & Lal, 2009; Recous et al., 1995).

NDRE values also indicated early mulch suppression, consistent with reduced canopy development due to limited nitrogen availability. At V10, non‐mulched treatments had 20% higher NDRE in 2022 and 14% higher in 2023. By R1, NDRE differences largely disappeared, though IFM showed the highest values in 2022, suggesting a potential interaction between mulch and nitrogen source. This response may reflect improved late‐season nitrogen availability in IFM, possibly due to reduced nitrogen losses or better moisture conservation under mulch.

Overall, plant growth responses closely followed early‐season nitrogen availability patterns observed in previous sections. Mulch consistently suppressed early vegetative growth by limiting emergence and immobilizing N, particularly in manure systems. However, these effects diminished in reproductive stages, demonstrating that corn can compensate when nitrogen becomes available later in the season. These results highlight the tradeoff between early growth suppression and improved nitrogen retention under mulch, particularly under SDI systems.

### 3.5 Agronomic parameters

Consistent with the nitrogen availability patterns, pore‐water nitrate dynamics, and plant growth responses described earlier, corn grain yield, HI, and PFP varied across treatments and years, reflecting tradeoffs between productivity and environmental outcomes (Table 2).

**TABLE 2:** Treatment means and significance for corn grain yield (GY), harvest index (HI), and partial factor productivity (PFP) as affected by treatments in 2022 and 2023, Concord, NE.

	2022			2023		
Treatments	GY (Mg ha^−1^)	HI	PFP (kg grain kg^−1^ N)	GY (Mg ha^−1^)	HI	PFP (kg grain kg^−1^ N)
MNM	15.6bc	0.49b	99bc	14.7a	0.55	67a
MM	14.8c	0.5b	94c	11.9b	0.56	54b
IFNM	17a	0.54a	108a	13.8a	0.55	62a
IFM	16.3ab	0.54a	104ab	11.8b	0.57	53b
**Contrasts**	*P* > F					
Manure vs. inorganic	**	***	**	NS	NS	NS
Mulch vs. No mulch	NS	NS	NS	**	NS	**

*Note*: MNM and MM indicate manure treatments without and with mulch, respectively; IFNM and IFM indicate inorganic fertilizer treatments without and with mulch, respectively. Treatment means followed by the same letters within a column are not significantly different. Columns that do not contain letters indicate no significant difference between treatments.

Abbreviation: NS, not significant.

*, **, and ***: Significant at *p* < 0.05, 0.01, and 0.001, respectively.

In 2022, inorganic fertilizer treatments (IFM and IFNM) produced the highest grain yields, averaging 16.7 Mg ha^−1^, about 9% greater than manure‐based treatments. This yield advantage corresponds with the greater early‐season nitrogen availability and enhanced vegetative growth observed in these treatments. This advantage was largely due to rapid early‐season N availability, which supported stronger vegetative growth. However, this came with higher environmental risk. Inorganic fertilizer plots, particularly IFNM, had elevated early‐season pore water nitrate concentrations (Figures 1 and 2), indicating a greater potential for nitrate leaching during rainfall or irrigation. The lack of mulch in IFNM increased early‐season nitrate availability and mobility, enhancing crop uptake and yield while also increasing the risk of nitrate leaching. These results align with earlier findings that rapid‐release fertilizers increase yield but compromise environmental protection (Abendroth et al., 2011; Woli et al., 2013).

In 2023, yields were lower overall, averaging 13.05 Mg ha^−1^. Less favorable weather, planting delays, and the use of a shorter‐maturity hybrid likely contributed to the decline. Fertilizer source did not significantly affect yield, but mulch reduced yields by about 17% in both manure (MM) and inorganic (IFM) treatments. Mulch likely delayed soil warming and slowed N mineralization, reducing early N availability (Mupangwa et al., 2007). While this reduced productivity, mulched treatments consistently lowered pore water nitrate concentrations and nitrate leaching, highlighting a tradeoff between yield and environmental outcomes.

The HI in 2022 was 8% higher in inorganic fertilizer treatments compared with manure, suggesting more efficient biomass partitioning into grain (Cassman et al., 2002). This advantage mirrored their yield benefit but again corresponded with greater nitrate losses. In 2023, HI values (0.55–0.57) did not differ among treatments, suggesting that environmental factors, rather than fertilizer source or mulch, dominated biomass allocation (Tollenaar & Lee, 2002).

The PFP in 2022 was also greater for inorganic fertilizer treatments, while manure treatments were 9% lower due to slower N release and reduced early‐season availability (Dobermann, 2005). In 2023, overall PFP declined and did not differ by fertilizer source, though mulch reduced PFP by 17%, again reflecting delayed N release (Ladha et al., 2005).

Overall, results indicate that inorganic fertilizers, particularly without mulch, maximized yield and PFP but at the cost of higher nitrate leaching. Conversely, manure and mulch reduced leaching but limited yield and NUE. These results highlight the importance of synchronizing nitrogen supply with crop demand to balance productivity and environmental sustainability under SDI systems.

### 3.6 Residual soil nitrogen

**FIGURE 5:**
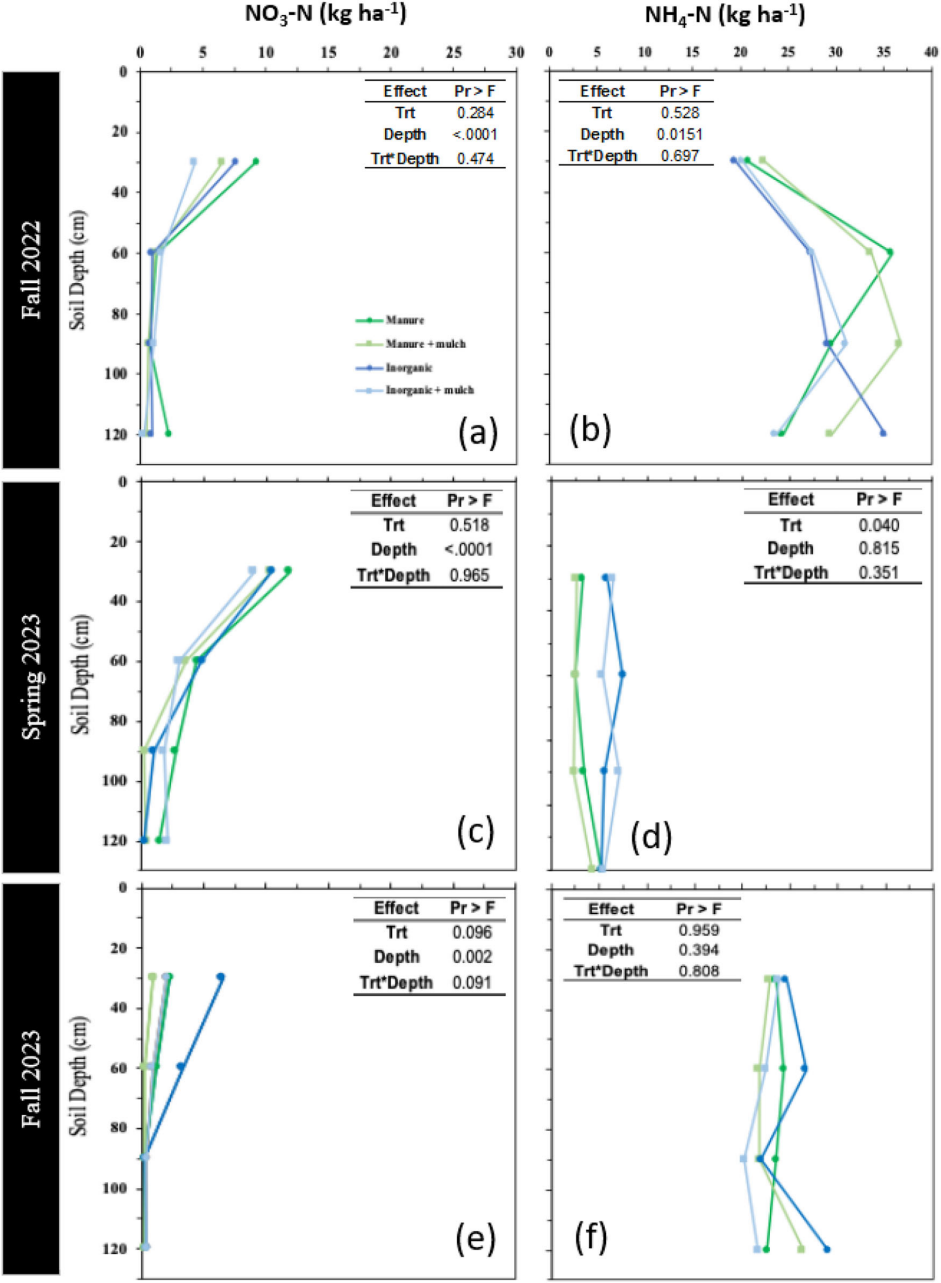
Mean residual soil nitrate‐N (NO_3_^−^‐N; left panels) and ammonium‐N (NH_4_^+^‐N; right panels) concentrations across the 0–120 cm soil profile as affected by fertilizer source and mulch treatments at Concord, NE. Values represent treatment means of replications (*n* = 4). Panels (a and b) show fall 2022 residual NO_3_^−^‐N and NH_4_^+^‐N; panels (c and d) show spring 2023 residual NO_3_^−^‐N and NH_4_^+^‐N; and panels (e and f) show fall 2023 residual NO_3_^−^‐N and NH_4_^+^‐N, respectively.

Residual soil N measurements provided insight into the fate of applied N under different fertilizer and mulch treatments. In fall 2022, residual soil NO_3_^−^‐N concentrations across the 0–120 cm profile were low, ranging from 8–14 kg N ha^−^^1^, with no significant differences between fertilizer sources or mulch treatments (Table 3). Depth effects were evident (Figure 5a), with about 70% of nitrate concentrated in the top 30 cm, consistent with the zone of active root growth and nutrient cycling (Di & Cameron, 2002). This vertical stratification highlights why early‐season pore‐water nitrate concentrations were elevated in non‐mulched treatments, as surface nitrate is more prone to leaching following rainfall or irrigation (Giri et al., 2026).

**TABLE 3:** Cumulative treatment means and significance for residual soil NO_3_^−^‐N and NH_4_^+^‐N across the 0–120 cm soil profile in fall 2022, spring 2023, and fall 2023 at Concord, NE.

	2022		2023			
Treatments	Fall (kg N ha^−1^)		Spring (kg N ha^−1^)		Fall (kg N ha^−1^)	
	NO_3_^−^‐N	NH_4_^+^‐N	NO_3_^−^‐N	NH_4_^+^‐N	NO_3_^−^‐N	NH_4_^+^‐N
MNM	14	110	33.4	14.3b	4.3	94.3
MM	10	122	15.4	11.6b	2.0	93.0
IFNM	11	110	14.9	25.4a	11.1	102.4
IFM	8	102	16.3	24.0a	3.9	88.6
**Contrasts**	*P* > F					
Manure vs. inorganic	NS	NS	NS	**	NS	NS
Mulch vs. No mulch	NS	NS	NS	NS	NS	NS

*Note*: MNM and MM indicate manure treatments without and with mulch, respectively; IFNM and IFM indicate inorganic fertilizer treatments without and with mulch, respectively. Treatment means followed by the same letters within a column are not significantly different. Columns that do not contain letters indicate no significant difference between treatments.

Abbreviation: NS, not significant.

** Significant at *p* <  0.01.

Residual NH_4_^+^‐N was considerably higher than nitrate in fall 2022, ranging from 102 to 122 kg N ha^−^^1^. No treatment differences were detected, but a depth effect was observed, with higher values deeper in the soil profile (Figure 5b). Ammonium in the profile likely originated from mineralization of soil organic matter. Lower NH_4_^+^ concentrations in the upper soil layers can be attributed to rapid nitrification and plant uptake during the growing season, which convert ammonium to nitrate or remove it from the soil system. In contrast, higher NH_4_^+^ concentrations at depth may reflect reduced nitrification rates due to lower microbial activity, cooler temperatures, and limited oxygen availability, allowing ammonium to persist in these layers (Cameron et al., 2013; Malhi et al., 2001).

By spring 2023, soil nitrate levels across the soil profile (0–120 cm) remained modest, averaging 20 kg N ha^−^^1^ and were concentrated in the upper 30 cm (Figure 5c), typical of post‐winter mineralization when microbial activity resumes (Randall & Mulla, 2001). Again, no treatment differences were detected. By fall 2023, nitrate declined further to an average of 5 kg N ha^−^^1^, largely confined to the surface layer (Table 3; Figure 5e), indicating crop uptake and potential nitrate leaching losses beyond the sampled soil profile following late‐season rainfall. In contrast, ammonium dynamics showed some treatment effects. In spring 2023, NH_4_^+^‐N concentrations were significantly higher in inorganic fertilizer plots (25 kg N ha^−^^1^) than in manure plots (13 kg N ha^−^^1^), with no clear depth stratification (Figure 5d). However, this elevated ammonium did not correspond with reduced nitrate leaching or improved crop uptake, as pore‐water nitrate concentrations and leaching losses remained greater in inorganic treatments. By fall 2023, NH_4_^+^‐N levels were similar across all treatments and depths (Table 3; Figure 5f), indicating stabilization of ammonium pools following seasonal nitrogen transformations.

Overall, residual soil N patterns indicate that inorganic fertilizers left higher ammonium in spring but were more prone to conversion and leaching as nitrate. In contrast, manure, particularly with mulch, promoted longer N retention in stable forms and reduced leaching losses. These findings underscore the potential of integrated organic amendments and surface residue management for sustaining crop productivity while minimizing environmental risk under SDI in sandy soils.

### 3.7 Nitrogen use efficiency

In 2022, grain nitrogen concentration averaged 1.4% across treatments with no significant differences (Table 4). Grain N uptake was also similar between fertilizer sources, indicating that both manure and inorganic fertilizer adequately supplied N during grain filling. However, mulch reduced grain N uptake by 9% compared with non‐mulched plots, likely reflecting early‐season immobilization from the high C:N ratio of woodchips. Aboveground biomass N uptake, in contrast, was influenced by fertilizer source: manure plots accumulated 11% more total biomass N than inorganic fertilizer plots, suggesting better synchronization between manure N release and crop demand. Mulch reduced biomass N uptake by 16%, possibly through cooler soil temperatures and slower mineralization (Gentry et al., 2009).

**TABLE 4:** Treatment means and significance for N concentration, uptake, and N harvest index (NHI) as influenced by fertilizer source and mulch treatments in 2022 and 2023, Concord, NE.

Treatments	2022				2023			
	Grain N conc (%)	Grain N uptake (kg N ha^−1^)	Aboveground biomass N uptake (kg N ha^−1^)	NHI (%)	Grain N conc (%)	Grain N uptake (kg N ha^−1^)	Aboveground biomass N uptake (kg N ha^−1^)	NHI (%)
MNM	1.3	223	346a	65b	1.2	158a	227a	70
MM	1.4	195	284bc	69b	1.2	129bc	182b	71
IFNM	1.4	208	303b	69b	1.2	150ab	219a	68
IFM	1.4	197	264c	75a	1.2	126c	180b	70
**Contrasts**	*P* > F							
Manure vs. inorganic	NS	NS	*	**	NS	NS	NS	NS
Mulch vs. No mulch	NS	*	***	*	NS	**	***	NS

*Note*: MNM and MM indicate manure treatments without and with mulch, respectively; IFNM and IFM indicate inorganic fertilizer treatments without and with mulch, respectively. Treatment means followed by the same letters within a column are not significantly different. Columns that do not contain letters indicate no significant difference between treatments.

Abbreviation: NS, not significant.

*, **, and ***: Significant at *p* < 0.05, 0.01, and 0.001, respectively.

In 2023, grain N concentration averaged 1.2% across treatments, again without differences among fertilizer sources (Table 4). However, mulch treatments (MM and IFM) had 17%–19% lower grain and biomass N uptake compared with their non‐mulched counterparts, consistent with reduced N availability during early growth. Non‐mulched treatments (MNM and IFNM) supported greater N uptake, likely due to faster mineralization and reduced immobilization (Malhi et al., 2001).

The NHI, the proportion of plant N allocated to grain, varied by treatment (Table 4). In 2022, NHI averaged 69% across all treatments. The IFM treatment recorded a 9% higher NHI than other treatments, suggesting improved late‐season N translocation when mulch was paired with inorganic fertilizer. This may reflect mulch's moderating effect on soil temperature and moisture, which sustained N availability later in the season (Ma et al., 2020; Zhang et al., 2018). In 2023, NHI averaged 70% across treatments, with no significant differences, indicating that environmental factors such as precipitation distribution, temperature, and growing season conditions affecting nitrogen uptake and remobilization likely overshadowed treatment effects (Jaynes et al., 2001).

Across both years, treatments without mulch (MNM and IFNM) consistently supported higher N uptake and efficiency, reflecting enhanced early‐season mineralization and reduced immobilization. However, these treatments also exhibited higher seasonal pore water nitrate concentrations (Figure 3a,b), particularly in IFNM, indicating greater leaching potential. In contrast, manure and mulch combinations reduced early‐season N availability, lowering uptake and efficiency but also limiting nitrate losses.

Overall, the results illustrate a clear tradeoff. Non‐mulched treatments, especially IFNM, maximized N uptake and N use efficiency but at the expense of higher leaching risk. Mulch and manure treatments improved N retention and environmental protection but reduced early uptake and yield potential. These outcomes highlight the challenge of balancing agronomic efficiency with groundwater protection in irrigated sandy soils.

## 4 CONCLUSIONS

This study highlights the critical role of nitrogen sources and mulch management in balancing agronomic performance with environmental stewardship in irrigated sandy soils of northeast Nebraska, a region representative of many vulnerable irrigated systems across the US Midwest. The results demonstrate that while inorganic nitrogen fertilizers effectively support early‐season corn growth and yield, they significantly increase the risk of nitrate leaching, especially in the absence of surface mulch. In contrast, manure‐based nitrogen management consistently reduced nitrate leaching losses and contributed to more stable soil nitrogen dynamics throughout the growing season. Although mulch alone offered moderate reductions in nitrate concentration peaks, its effectiveness was substantially enhanced when paired with inorganic fertilizer. This integrated approach minimized environmental nitrogen losses, although some yield reductions were observed under mulch, particularly in years with delayed early‐season growth.

Overall, these findings underscore the importance of site‐specific nitrogen management strategies that replace or supplement inorganic fertilizers with organic sources like manure or surface mulch. Such practices enhance NUE and reduce nitrate leaching, contributing to both agronomic productivity and groundwater protection. Integrating manure and mulch into conventional inorganic nitrogen fertilizer management supports the principles of the 4Rs of nutrient stewardship and offers a practical, sustainable path forward for producers managing irrigated sandy soils in nitrate‐sensitive regions.

## AUTHOR CONTRIBUTIONS

**Swetabh Patel**: Conceptualization; data curation; formal analysis; methodology; validation; visualization; writing—original draft; writing—review and editing. **Michael Kurtzhals**: Data curation; investigation; writing—review and editing. **Arshdeep Singh**: Data curation; formal analysis; validation; visualization; writing—review and editing. **Amy M. Schmidt**: Conceptualization; data curation; funding acquisition; investigation; methodology; project administration; resources; supervision; validation; visualization; writing—review and editing. **Leslie Johnson**: Conceptualization; investigation; methodology; supervision; writing—review and editing. **Javed Iqbal**: Conceptualization; data curation; formal analysis; funding acquisition; investigation; methodology; project administration; resources; software; supervision; validation; visualization; writing—review and editing.

## CONFLICT OF INTEREST STATEMENT

The authors declare no conflicts of interest.

## Supporting information



Supplementary tables and figures
