# Supplementary material for: Fertilizer source and mulch effects on nitrate‐N leaching and corn yield in irrigated sandy loam soil
Source: J Environ Qual. 2026 Aug 5;55(4):e70226. doi: 10.1002/jeq2.70226 (PMC13443348; doi:10.1002/jeq2.70226)
Supplement: Supplementary file 1 [file JEQ2-55-0-s001.docx]

**Supplementary tables and figures:**

Table S1. Soil information and initial soil test values (0–20 cm) for the study site at Concord, NE.

| Property | Value |
| --- | --- |
| Soil pH (1:1 soil to water) | 6.0 ± 0.2 |
| Soil Organic Matter (%) | 2.3± 0.3 |
| NO_3_-N (mg kg^-1^) | 9 ± 1 |
| Mehlich-III P (mg kg^-1^) | 17 ± 3.1 |
| SO_4_ (mg kg^-1^) | 9 ± 1 |
| K (mg kg^-1^) | 143 ± 7 |
| Ca (mg kg^-1^) | 1230 ± 97 |
| Mg (mg kg^-1^) | 168 ± 16 |
| Na (mg kg^-1^) | 8 ± 1 |
| Sum of Cations (me 100g^-1^) | 11.4 ± 1.1 |
| Soil Texture | Sandy Loam |

Table S2. Field operations (tillage, fertilizer, herbicide, mulch), hybrid used, seeding rate, planting, and harvest of corn during the study conducted from 2022-2023 at Concord, NE.

| **Field Operation** | **2022** | **2023** |
| --- | --- | --- |
| Tillage | No-till | No-till |
| Total fertilizer applied | 140 kg N ha^-1^ | 197 kg N ha^-1^ |
| Woodchips | 37 t ha^-1^ | 48 t ha^-1^ |
| Planting date | 11^th^ April | 18^th^ May (re-planted on 7^th^ June) |
| Hybrid | P1108Q (111 RM) | P0339AM (103 RM) |
| Seeding rate | 81000 kernels ha^-1^ | 81000 kernels ha^-1^ |
| Harvest | 11^th^ October | 23^rd^ October |

Precipitation (mm)

|  | 2022 | 2023 | Long-term Average |
| --- | --- | --- | --- |
| Apr | 18.796 |  | 66.04 |
| May | 76.454 | 95.504 | 106.147 |
| Jun | 33.274 | 114.046 | 134.137 |
| Jul | 64.008 | 41.402 | 52.6542 |
| Aug | 37.338 | 0 | 94.7674 |
| Sep | 18.034 | 0 | 65.0494 |
| Oct | 17.018 | 0 | 62.4078 |

Figure S1: Monthly precipitation during the corn growing season in each site-year of the study, and 20-years long-term (2002-2021) precipitation average for the study site.
